# Sex Differences in Processing Emotional Speech Prosody: Preliminary Findings from a Multi-Feature Oddball Study

**DOI:** 10.3390/brainsci14121216

**Published:** 2024-11-30

**Authors:** Chieh Kao, Yang Zhang

**Affiliations:** Department of Speech-Language-Hearing Sciences, University of Minnesota, Minneapolis, MN 55455, USA; kaoxx096@umn.edu

**Keywords:** emotional prosody, multi-feature oddball, MMN, P3a, sex differences

## Abstract

Background/Objectives: Emotional prosody, the intonation and rhythm of speech that conveys emotions, is vital for speech communication as it provides essential context and nuance to the words being spoken. This study explored how listeners automatically process emotional prosody in speech, focusing on different neural responses for the prosodic categories and potential sex differences. Methods: The pilot data here involved 11 male and 11 female adult participants (age range: 18–28). A multi-feature oddball paradigm was used, in which participants were exposed to sequences of non-repeating English words with emotional (angry, happy, sad) or neutral prosody while watching a silent movie. Results: Both mismatch negativity (MMN) and P3a components were observed, indicating automatic perceptual grouping and neural sensitivity to emotional variations in speech. Women showed stronger MMN to angry than sad prosody, while men showed stronger MMN to angry than happy prosody. Happy prosody elicited the strongest P3a, but only in men. Conclusions: The findings challenge the notion that all facets of emotion processing are biased toward female superiority. However, these results from 22 young adult native English speakers should be interpreted with caution, as data from a more adequate sample size are needed to test the generalizability of the findings. Combined with results from studies on children and elderly adults, these preliminary data underscore the need to explore the complexities of emotional speech processing mechanisms to account for category and sex differences across the lifespan in a longitudinal perspective.

## 1. Introduction

In daily communication, listeners rely on both the content and style of speech to fully understand the spoken message. Emotional prosody is a stylistic register characterized by variations in key acoustic parameters such as pitch, intensity, and timing, and these prosodic manipulations can significantly alter the interpretation of a sentence [1]. When there is a discrepancy between the emotional tone and the words, listeners tend to rely more on the tone [2,3,4,5,6,7].

To study how listeners perceive rapidly changing emotional prosody, electroencephalography (EEG) has been frequently used in previous studies [8]. By averaging tens or hundreds of trials of time-locked EEG responses to the stimuli to obtain the event-related potentials (ERPs), studies have used simple speech elements like vowels [9], syllables [10,11,12], or fixed words [13,14,15] to convey emotions. These studies have primarily used the auditory oddball paradigm [16]. In this paradigm, a standard sound is typically presented most of the time (e.g., 80% of the trials) while a deviant sound is randomly interspersed. The mismatch negativity (MMN) and P3a components are commonly observed in ERPs to emotional prosody changes [9,11,15,17,18]. The MMN response peaks at approximately 150~200 ms after the onset of acoustic change in the deviant sound relative to the standard sound, and it appears as a negative deflection at the centro-frontal electrodes [10,12,13,14]. The MMN indexes automatic neural processing that occurs in response to changes in the environment, even before conscious attention is directed to the stimulus. While initially linked to acoustic discrimination, the MMN was later observed to have stronger responses to prosodic changes in real words compared to pseudowords, suggesting higher-level cognitive processing at this early, pre-attentive stage [10,15]. Following the MMN, P3a is a positive deflection elicited around 350 ms after the acoustic change onset, and it is fronto-centrally oriented over the scalp [11,15,18,19]. The P3a is linked to cognitive evaluations of the incoming sounds and the involuntary attention switch to the novel auditory input [16,20,21,22,23]. P3a can be elicited by voice changes from neutral to affective prosody [9,17], reflecting the allocation of attention to unexpected or emotionally salient events and making it particularly relevant for studies examining emotional prosody. For instance, Zora et al. [15] demonstrated that P3a responses were stronger to emotional prosody than non-emotional prosody (i.e., word stress), indicating that P3a is sensitive to affective salience beyond just the acoustic processing.

However, it remains unclear how the brain processes emotional prosody across diverse speech stimuli, as encountered in everyday life. To date, the MMN and P3a findings have mainly tested two emotional prosodies (one as the standard and the other as the deviant), with a limited exploration of neural responses to multiple vocal emotional categories [9]. Concerns have been raised regarding the use of simple stimuli and the generalizability of findings [15]. To compare the neural responses to multiple emotional categories, we turned to the multi-feature oddball paradigm [24,25]. The multi-feature oddball paradigm limits the presentation of the standard sound to 50% and allows different types of deviants to equally take up the remaining 50%. To address the issue of generalizability, we introduced a further modification by including non-repeating spoken words for each emotional prosody and demonstrated its feasibility [26,27]. 

One important finding in emotional perception concerns sex differences [12,28,29,30,31,32,33]. For instance, female listeners have been found to recognize subtle emotional tones better, and they are more susceptible to conflicting semantic information from the prosodic domain [34]. Similarly, ERP studies have shown that women tend to exhibit stronger mismatch negativity (MMN) responses to emotional prosody changes compared to men [10,35]. However, in acoustic control conditions without involving speech, sex differences in the MMN disappeared [10,11,36]. By comparison, sex effects on P3a response to vocal expressions of emotion have rarely been reported. The simple setup using fixed stimuli may have masked potential sex differences in involuntary attentional responses to prosodic changes. Nevertheless, Hung and Cheng [11] observed a larger P3a response to emotional prosody changes in women compared to men. In another study, Garcia-Garcia et al. [37] used visual distractors to induce emotional context while participants passively listened to emotional voices and reported that only female listeners’ P3a amplitudes were influenced by the emotional context. These reports motivate further investigation into potential sex effects in MMN and P3a differences to multiple emotional voices.

The current study aimed to investigate the MMN and P3a components elicited by three emotional prosodic deviants (angry, happy, and sad) in a multi-feature oddball task using non-repeating spoken words, requiring the listeners to extract paralinguistic categories across varying lexical items. Given previous findings of stronger MMN and P3a responses to high-arousal negative emotions [9,11], we expected the strongest MMN and P3a responses to the angry deviant. While making highly specific hypotheses for each emotional voice may be challenging regarding potential sex differences, our exploratory analysis provides an initial test on whether the “female advantage” with stronger MMN and P3a responses can be observed in all or some of the three emotional prosodic changes. The term “female advantage” has been used in the literature to describe more efficient or enhanced neurophysiological processing (e.g., larger MMN and P3a responses) in females to emotional cues. We will use “female advantage” in the discussion of our current data in relation to the existing studies. However, this should be interpreted with caution, as the neural responses observed here reflect passive listening to emotional prosody, rather than performance in behavioral judgments of emotional voices.

## 2. Materials and Methods

### 2.1. Participants

The participants for this pilot study, which was based on data from a larger research program originally reported in a doctoral dissertation completed during the COVID-19 pandemic [38], were 22 monolingual native speakers of American English studying at the University of Minnesota. The sample consisted of 11 female and 11 male participants, all right-handed, aged between 18 and 28 years (mean age = 20.8), and all were Caucasian with no diagnosis of hearing- or language-related differences. They all had normal or corrected-to-normal vision. The experimental protocol was approved by the Institutional Review Board at the University of Minnesota. Participants signed the informed consent forms before the experiment, and each received USD 10 upon completion. 

### 2.2. Stimuli

All speech stimuli were taken from the Toronto Emotional Speech Set (TESS) [39], which includes 200 monosyllabic phonetically balanced words (Northwestern University Auditory Test No. 6, NU-6) [40]. The emotional categories in this publicly accessible database—happy, angry, sad, and neutral—have been validated to ensure clear distinctions between positive and negative valence, with prior behavioral studies confirming the effective conveyance of these emotions. Each of the 200 words was spoken in neutral, happy, sad, and angry voices by a young female speaker, yielding a total of 800 stimuli. The sounds were sampled at 24,414 Hz, with the mean sound intensity levels equalized at 70 dB SPL using PRAAT 6.0.40 [41]. Table 1 summarizes the mean fundamental frequency (F0), duration, intensity variation, harmonics-to-noise ratio (HNR), and spectral centroid in each emotional prosody. These five acoustic measures are commonly used to characterize different vocal emotions [1,42,43,44,45].

### 2.3. Procedure

We adopted a multi-feature oddball paradigm (or optimal paradigm) [14,24,25] to examine listeners’ early neural sensitivities to happy, angry, and sad prosodies compared to the neutral prosody. The multi-feature oddball paradigm is a passive listening protocol that allows us to measure three emotional prosody contrasts (from neutral to angry, from neutral to happy, and from neutral to sad) within the same recording session. We presented 600 trials in total. The Standard stimuli were words in a neutral tone (presented with 50% probability, 300 trials). 

**Figure 1:**
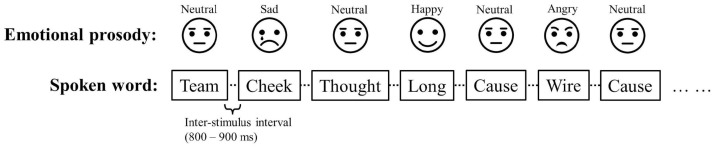
A schematic example of the order of the trials. The standard (neutral prosody) and deviant (angry, happy, and sad prosodies) were always alternating, and the three emotions (deviants) were pseudo-randomly interspersed.

The three emotional tones (happy, angry, sad) served as three types of deviant stimuli (each presented with 16.7% probability, 100 trials). For the 300 standard trials, all 200 words in the neutral voice were used and 100 words were randomly selected for repetition. For each type of the 100 deviant trials, 100 words in each emotional voice were randomly selected and used. The sounds were always presented in an alternating standard and deviant fashion. The three types of deviant (three emotions) were pseudo-randomly interspersed, with no consecutive deviant trials in the same emotional prosody (see Figure 1 for an example of the sound presentation order). The inter-stimulus interval (ISI) was randomized between 800 and 900 ms, and the total recording time was around 25 min.

Participants were seated in an electrically and acoustically treated booth (ETS-Lindgren Acoustic Systems, Austin, TX, USA) with a 64-channel WaveGuard EEG cap (ANT Neuro North America, Philadelphia, PA, USA). They were instructed to ignore the speech sounds and focus on a silent movie while the continuous EEG signals were recorded. The speech sounds were played via two loudspeakers (M-audio BX8a) placed at a 45-degree azimuth angle, 3 feet away from the participants and presented at 55 dB SL relative to the individual listener’s hearing threshold at 1 kHz [46]. The sound presentation was controlled by E-Prime (Version 2.0; Psychological Software Tools, Inc., Sharpsburg, PA, USA) using a Dell PC outside the sound-treated room. Continuous EEG data were recorded through the Advanced Neuro Technology EEG System (Advanced Source Analysis version 4.7). The WaveGuard EEG cap had a layout of 64 Ag/AgCl electrodes following the standard International 10–20 Montage system with intermediate locations, and it was connected to a REFA-72 amplifier (TMS International BV, Oldenzaal, The Netherlands). The bandpass filter for raw data recording was set at 0.016–200 Hz, and the sampling rate was 512 Hz. The electrode AFz served as the ground electrode. The impedances of all electrodes were kept under 5 kΩ.

### 2.4. Data Analysis

The preprocessing of EEG data was completed offline using EEGLAB v14.1.1 [47]. The continuous EEG data were low-pass filtered at 30 Hz, downsampled to 250 Hz, and high-pass filtered at 0.5 Hz. The EEG data were then re-referenced to the average of the two mastoid electrodes. Next, we applied the “Clean_rawdata” package to remove low-frequency drifts and non-brain activities (e.g., muscle activity, sensor motion, etc.). Data were then decomposed by the Independent Component Analysis (ICA) algorithm [48,49] to remove eye-blink artifacts. ERP epochs were extracted from 100 ms pre-stimulus onset to 1000 ms post-stimulus onset, and baseline correction was applied using the mean voltages of the 100 ms baseline period. Epochs containing data points exceeding the range of 100.0 μV were rejected before averaging. The numbers of trials remaining for each emotional prosody were 276 for neutral (standard), 89 for happy (deviant), 91 for angry (deviant), and 91 for sad (deviant). Using ERPLAB v7.0.0 [50], averaged event-related potentials (ERPs) were derived for standard (neutral prosody) and all three types of the deviants (angry, happy, and sad prosodies). Difference waveforms were created by subtracting the standard ERP from each deviant ERP, yielding happy, angry, and sad difference waveforms. Neutral prosody served as the standard stimulus for deriving the MMN and P3a components for detecting changes in emotional prosody (happy, angry, and sad) from neutral and was not analyzed as a separate condition in the model, as it was used as the reference for comparison.

All statistical analyses were completed in R (https://www.r-project.org/) with the packages “lme4” [51], “lmerTest” [52], and “emmeans” [53]. The difference waveforms were used for assessing the target components MMN (200–300 ms) and P3a (350–450 ms). The time window for each component was selected based on previous neurophysiological reports of emotional prosody perception [14,15,17] and visual inspection of the current grand average difference waveforms. The amplitudes of MMN and P3a for statistical analyses were calculated as the mean voltages of the 40 ms peak (20 ms before and after the peak value) of the difference waveforms within the two time windows (MMN, 200–300 ms; P3a, 350–450 ms). The most negative 40 ms peak value was extracted for MMN, and the most positive 40 ms peak for P3a. Peak amplitude extraction was applied to channels at frontal (F-line, F3, Fz, F4), central (C-line, C3, Cz, C4), and parietal (P-line, P3, Pz, P4) regions [15]. These peak amplitudes were then used as the dependent variables in the following statistical models. 

Linear mixed-effect models were separately implemented on MMN and P3a amplitudes. Each model included by-participant intercept as a random-effect factor. Emotion (happy, sad, and angry), region of the electrode (anterior, central, and parietal), and laterality of the electrode (left, middle, and right) were included as trial-level fixed-effect factors. Sex (female and male) was included as a participant-level fixed factor. Finally, cross-level interactions of emotion and sex were also included. Post hoc *t*-tests with Bonferroni corrections (α = 0.05) were performed to characterize any significant interaction. 

## 3. Results

**Figure 2:**
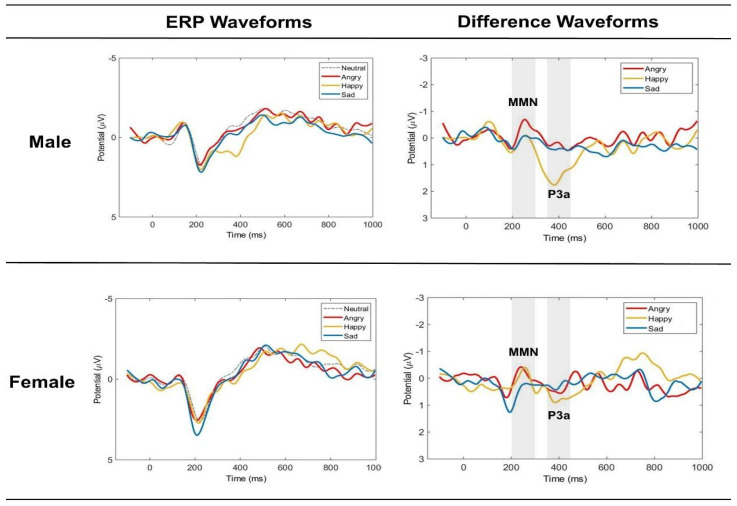
The grand mean event-related potential (ERP) waveforms of standard (neutral prosody) and deviants (angry, happy, and sad), and grand mean difference waveforms of angry, happy, and sad for male and female listeners. Mean amplitudes of the midline electrodes (Fz, Cz, Pz) were used for the waveforms. The gray shaded areas mark the windows for MMN (200–300 ms) and P3a (350–450 ms).

**Figure 3:**
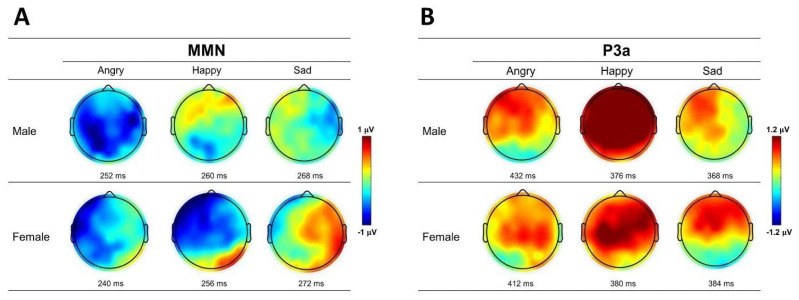
The scalp topographic maps of (**A**) MMN and (**B**) P3a to angry, happy, and sad emotional prosodies averaged across male and female listeners. The topographies are based on the latencies of peak values at Cz channel.

The linear mixed-effect models allowed for an in-depth analysis of potential contributors to the MMN and P3a amplitude measures extracted from the difference waveforms, which were derived by subtracting the standard ERP from the deviant ERPs. Both female and male listeners showed distinct MMN and P3a peaks to the change in emotional prosody, and their grand mean ERP waveforms and difference waveforms were recorded from midline electrodes (Fz, Cz, Pz) for all emotional prosodies (Figure 2). The topographic maps of MMN and P3a peaks of each emotional prosody are presented in Figure 3.

### 3.1. Mismatch Negativity (MMN)

**Figure 4:**
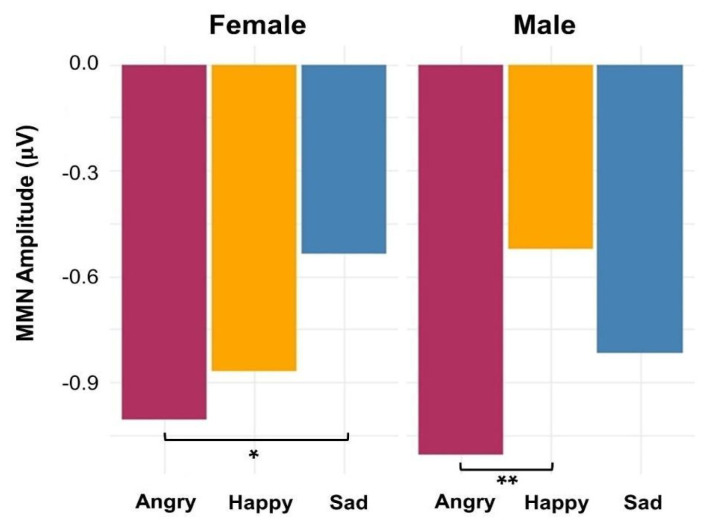
The interaction effect of emotion and sex displayed in the model predicted MMN amplitudes to angry, happy, and sad emotional prosodies in male and female listeners. (* stands for *p* < 0.05; ** for *p* < 0.01).

The main effect of emotion (*F*(2,572) = 8.21, *p* < 0.001) was significant, but not the main effects of electrode region (*F*(2,572) = 1.27, *p* = 0.28) or electrode laterality (*F*(2,572) = 0.59, *p* = 0.55). In general, MMN to angry prosody was stronger than happy (*p* = 0.002) and sad prosodies (*p* = 0.001). The participant-level main effect of sex was not significant (*F*(1,22) = 0.0013, *p* = 0.97), but there was a significant interaction between emotion and sex (*F*(2,572) = 4.68, *p* = 0.009). Post hoc *t*-tests revealed that male listeners showed stronger MMN to angry than happy prosody (*p* = 0.002), whereas female listeners showed stronger MMN to angry than sad prosody (*p* = 0.02). The model results are summarized in Table 2, and Figure 4 shows the interaction effect of emotion and sex on MMN amplitudes. The model marginal *R*^2^ was 0.04, indicating that 4% of the variance was explained by the fixed effects alone; the model conditioned *R*^2^ was 0.35, indicating that 35% of the variance was explained by both fixed and random effects. These results suggest that individual variability plays an important role in explaining the observed outcomes. Given the relatively small effect size for the fixed effect variables and the small sample size for this pilot study, the reported significant *p*-values should be interpreted with caution and will need to be further verified with a larger sample size. 

### 3.2. P3a Component

**Figure 5:**
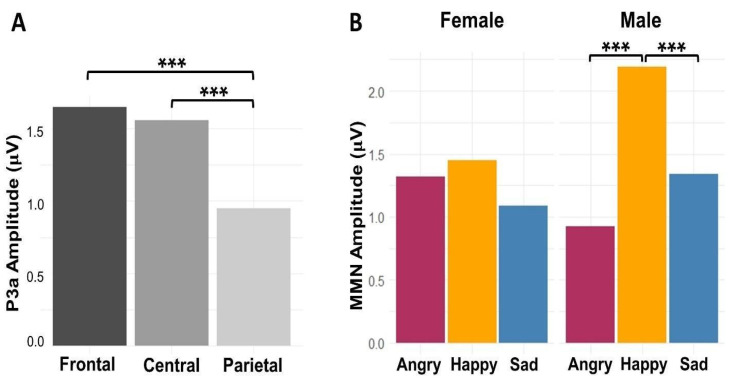
The (**A**) main effect of electrode region and (**B**) interaction effect of emotion and sex displayed in the model predicted P3a amplitudes to angry, happy, and sad emotional prosodies in male and female listeners. (*** stands for *p* < 0.001).

The main effects of emotion (*F*(2,572) = 25.67, *p* < 0.001) and electrode region (*F*(2,572) = 26.13, *p* < 0.001) were significant, but not the main effect of electrode laterality (*F*(2,572) = 1.54, *p* = 0.21). In general, P3a to the happy prosody was stronger than angry and sad prosodies (*ps* < 0.001), and frontal channels and central channels recorded stronger P3a than parietal channels (*ps* < 0.001). The participant-level main effect of sex was not significant (*F*(1,22) = 0.49, *p* = 0.49), but there was a significant interaction between emotion and sex (*F*(2,572) = 14.61, *p* < 0.001). Post hoc *t*-tests revealed that male listeners showed stronger P3a to happy than angry (*p* < 0.001) and sad (*p* < 0.001) prosodies, whereas female listeners showed similar P3a to all emotional prosodies. The model results are summarized in Table 3, and Figure 5 shows the main effect of electrode region and the interaction effect of emotion and sex on P3a amplitudes. The model marginal *R*^2^ was 0.13, indicating that 13% of the variance was explained by the fixed effects alone; and the model conditioned *R*^2^ was 0.37, indicating that 37% of the variance was explained by both fixed and random effects. While the effect size for the fixed effects here increased to represent a moderate level in comparison with that for MMN, the overall trend remains the same with individual differences (captured by random effects), being a key contributor to the findings. 

## 4. Discussion

Emotional prosody in speech plays a vital role in social communication, yet it has not received as much attention as visual emotional cues in research. Understanding how listeners interpret rapidly changing emotional cues is crucial for developing models of language processing that incorporate both semantic and prosodic information. This study used a multi-feature auditory oddball paradigm to investigate listeners’ neural responses to emotional prosody changes. Unlike prior studies with limited lexical content, we used continuously changing stimulus presentations with varying spoken words to deliver emotional prosodies. Our results show that listeners could extract emotional prosodic information from non-repeating spoken words, as evidenced by MMN and P3a responses to angry, happy, and sad voices. These findings suggest that abstract emotional prosodic categories based on perceptual grouping can be tested in naturally changing speech stimuli, even with high acoustic variations. Importantly, testing three emotional deviants in one task did not compromise the target ERP components for emotional prosodic change detection. Our results support previous findings that MMN reflects change detection at a higher-level category [13,15,16,54]. Additionally, we found emotion-specific and sex-specific effects in the MMN and P3a responses.

### 4.1. MMN—Angry Voices Elicited the Strongest Response in Both Men and Women

A stronger MMN response was observed when the background voice changed from neutral to angry compared to other emotional categories, indicating a heightened automatic processing of high-arousal negative emotional sounds, even without attention. This enhanced response, occurring around 200 ms after sound onset, suggests the early activation of pre-attentive sensory processing when affective signals change. Such a “negative bias” response to emotions like anger or fear is considered crucial for survival due to their association with immediate threats or danger [35,55,56]. Our results align with the notion of negativity bias response [9] and support the idea that the pre-attentive system quickly checks and responds to category contrasts in affective voices, particularly those signaling potential threats.

Clear sex differences were observed in the ERP measures. Females showed stronger MMNs to angry than sad voices, while males showed stronger MMNs to angry than happy voices. Women’s early neural responses distinguished angry from sad but not from happy voices, whereas men’s response distinguished angry from happy but not from sad voices. Female listeners did not exhibit stronger MMN than males in any emotional deviants, contrary to previous studies showing enhanced emotional MMN in women [10,11]. The ERP waveform plot (Figure 2) showed women’s indistinguishable MMN to angry and happy sounds was mainly due to their enhanced MMN for happy prosody, aligning with their greater automatic processing of happy facial expressions [57], indicating their perceptual advantage of positive emotion processing even at the pre-attentive level. In contrast, men showed a more distinct MMN to angry prosody than the other emotions, suggesting heightened early neural sensitivity to angry voices (Figure 2). Here, the preserved pre-attentive processing sensitivity to angry prosody in our male participants is consistent with previous behavioral studies showing their heightened response to angry emotional information [58]. These results suggest sex-specific adaptive strategies in how males and females automatically extract and respond to emotional prosody information, even at the pre-attentive level.

### 4.2. P3a—Happy Voices Elicited the Strongest Response, Especially in Men

The P3a response in our study was fronto-centrally oriented, consistent with the topographic distribution of the classical P3a [23,59]. Statistical results confirmed the significant main effect of the electrode region. As a later neural response after the MMN, the P3a indicates an involuntary attentional shift to novel auditory input and involves signal appraisal. Our listeners, particularly males, exhibited the strongest P3a to the happy voice among the three emotional deviants. Previous studies have mainly reported enhanced P3a to general affective information, with few examining P3a differences for individual emotional prosodies [13,14,15,17]. One study [60] used laughter and growl to present happy and angry voices, and they asked participants to pay attention to the sounds during the EEG recording. Their results showed enhanced positive deflection to laughter at 350–450 ms after the sound onset, similarly to our P3a timing. Another report presented different emotional prosodies over French vowels and observed stronger P3a to the happy voice than sad and neutral voices [9]. Our results, showing increased P3a to happy prosody over non-repeating spoken words, are consistent with these findings, suggesting an involuntary attentional bias toward positive information after initial sensory processing (i.e., MMN).

Sex differences in the P3a response were evident. However, contrary to the “female advantage” prediction, they were primarily driven by male listeners. Women did not exhibit distinguishable P3a responses to the three emotional deviants. P3a is known to habituate, with its amplitude declining as listeners become more accustomed to deviant events [61]. It is possible that arousal and involuntary orienting to affective signals in female participants show similar habituation effects across different emotional prosody categories, leading to indistinguishable P3a responses. Given that our stimuli only included a female voice, one might expect to observe more pronounced sex differences in P3a in favor of women due to the influence of “in-group advantage” [62]. This concept suggests that individuals tend to process information from their own gender more efficiently. However, the P3a data in our study revealed that this was not the case. As most studies on P3a and emotional prosody change did not examine sex effects [9,15,17], further research is needed to understand the functional significance of P3a and its association with subsequent behavioral responses with regard to sex differences in this late neural sensitivity marker. 

### 4.3. Comparison with the Behavioral Studies in the Literature

Recent behavioral studies with larger groups of participants showed mixed results on sex differences in identifying emotional information. One study with over 100,000 participants across generations found that female participants showed better facial emotion recognition, but the performance gap between female and male participants decreased with age [63]. A similar female advantage was reported in identifying emotional information in voices [64], and female listeners’ better emotional speech recognition was less evident in the older age group [32]. On the contrary, another study with a community sample of more than 5000 people did not support female advantage in recognizing emotions in faces [65]. In our ERP data collected from a passive listening condition, the lack of a clear across-the-board “female advantage” in the MMN and P3a response suggests that the relationship between sex and emotional processing may not be as straightforward as previously thought. It also points to the possibility that individual differences, such as prior experience, cognitive strategies, or socialization, could play a more significant role in emotional recognition than biological sex alone. This further supports the need for more refined research, especially considering how pre-attentive processing (like MMN and P3a) might be influenced by a variety of factors beyond sex, such as age, personality, cultural context, or other demographic variables.

### 4.4. Limitations and Future Directions

There are several limitations in our study. First, the emotional speech set of phonetically balanced words in the original database only contains female-voice recordings [39]. One neurophysiological study demonstrated that listeners showed early neuro-differentiation of emotional prosody information regardless of the speakers’ sex [30]. However, mixed patterns of behavioral results have been reported regarding the effects of speaker’s sex and the related stereotypes [64]. A recent behavioral study observed a modulatory effect of encoder sex of the speech stimuli on listeners’ emotional prosody recognition [34]. In this regard, including both female and male emotional voices, the stimuli can provide a more fine-grained view on listeners’ neural sensitivities to natural emotional speech prosody and the potential influences of the sex of the speaker. 

Second, we incorporated non-repeating real words to create a more natural linguistic context for delivering emotional prosody. Even though we carefully selected a phonetically balanced word list to control phonetic-level acoustic variations across emotional voices, the paralinguistic features such as pitch, intensity variation, and word durations still co-vary with different emotional prosodies. Isolating each acoustic feature in emotional voices for testing may not be realistic, as emotional prosody encompasses a combination of relevant acoustic properties [1,43,66]. One solution is to create four oddball tasks and use each of the neutral, happy, angry, and sad prosodies as the standard sound, and compare standard and deviant sounds of the same emotion across tasks. This solution may not be the most optimal one because it contradicts our purpose to establish an efficient testing protocol to record MMN and P3a to multiple emotional deviants that could potentially be applied to clinical and pediatric populations without requiring focused attention and extended hours of EEG recording. Nonetheless, a follow-up study with several multi-oddball recording sessions will still be valuable to verify the findings on the MMN and P3a components with the three emotional voices measured in the current study. 

Our modified multi-feature oddball protocol and findings contribute to the existing literature on neural sensitivities to emotional prosody, which has used a wide range of stimuli such as vowels [9], simple syllables [10,11,17,35,67] limited numbers of words [13,14], or non-speech sounds [68]. Collectively, these findings not only support the feasibility of the neurophysiological approach but also provide detailed insights into how the human pre-atten71tive system detects incoming emotional prosody changes in speech and triggers the involuntary attentional system in early stages of emotional prosody processing, including sex differences. These findings have important implications for future developmental and clinical studies [69,70,71,72].

As no behavioral responses were required in the passive listening paradigm, one could question whether the MMN and P3a responses were primarily driven by acoustic grouping or perception of the emotional categories. The opposite sex difference patterns in MMN and P3a for the angry and happy prosodies observed in our study suggest that the responses cannot be solely attributed to lower-level responses to acoustic change detection. A close examination of the stimulus properties (Table 1) showed that the largest mean fundamental frequency deviation was found in the happy prosody (15.9%), the largest mean duration deviation in the sad prosody (23.2%), and the largest intensity variation deviation in the angry prosody (2.1 dB), compared to the neutral prosody. Based on the MMN results from the multi-featured oddball study by Pakarinen et al. (2009) [25], a larger proportion of difference in duration (sad prosody relative to neutral prosody) would be expected to elicit a greater MMN response compared to a smaller proportion of differences in frequency and intensity for the two other deviants. However, in our study, the sad prosody with 23.2% deviation in duration actually produced the smallest MMN response. As the combined proportion of acoustic deviations of each of the angry, happy, and sad prosodies relative to the neutral prosody was far beyond just the noticeable differences [73], there would be no reason to expect highly significant sex differences for detecting easily distinguishable acoustic deviations. These considerations led to our alternative interpretation that other factors such as higher-level processing related to emotional perception or cognitive interpretation would have influenced the neural responses. 

In addition to examining emotional prosody processing in normal adults, recent studies highlight the importance of understanding the dynamic changes, including sex differences, in emotion processing across the lifespan, including infancy and aging. For instance, an earlier behavioral study showed that adolescent females tended to be more sensitive to happy and sad prosody compared to males, but not to angry prosody [74], and the researchers suggested that sex differences in emotional prosody recognition may begin to emerge during adolescence when there are distinct testosterone levels between males and females. However, our new infant study [27] using exactly the same multi-feature MMN paradigm and stimuli as the current study found significant age and sex differences in infants’ neural sensitivity to emotional prosodies, which did not mirror the adult effects observed in this report. Lin et al. [74] adopted a Stroop-like behavioral protocol and reported category-sensitive, age-related shifts between prosodic and semantic dominance in emotion perception, which were linked to cognitive capacities. Furthermore, Lin et al. highlighted that age and sex differences in emotion perception are influenced by emotional category and communication channel. These findings demonstrate that the processing of emotional prosody is not static; rather, it evolves through developmental stages. Aging populations also face challenges in affective prosody recognition [75,76]. A recent systematic review and meta-analysis by Fan et al. [77] highlighted a positivity bias among older adults in contrast to the negativity bias typically observed in younger adults. Together, these studies emphasize the importance of adopting a longitudinal perspective to fully understand the dynamics of emotional prosody in multimodal speech communication across the lifespan.

Given the small sample size in our study, it is premature to determine the reliability of sex differences in terms of how males and females are capable of capturing the change in incoming abstract emotional prosody categories differently in a highly dynamic speech context at the pre-attentive stage. To address this concern further, additional acoustic control experiments with a much larger and more diverse sample size will need to be introduced in future studies by testing stimuli with similar acoustic properties to the emotional prosody but without the vocal quality as well as the speech content. Simultaneous behavioral and ERP measures with an attentive listening condition that produces P3b responses would also help clarify how discriminable the emotions in the spoken words are and the extent to which the observed MMN and P3a responses and the associated sex differences are driven by acoustic features versus emotional perception.

## 5. Conclusions

Using a passive listening paradigm with EEG recording, we assessed listeners’ pre-attentive neural sensitivity in extracting and discriminating affective prosodic categories across hundreds of spoken words and the subsequent involuntary orientation to prosodic contrasts without overt behavioral reactions. Despite the limited sample size and exploratory nature, the MMN and P3a results confirmed the feasibility of our modified multi-feature oddball task, with pilot data showing significant category and sex differences in emotion processing outside of attentional focus. This paradigm provides a new protocol for future studies on emotional prosody with potential for extension from adults to infants, children, and individuals with difficulties in affective processing. Future work with an increased sample size and diversity is needed to investigate sex differences in social and cognitive abilities [78,79] and elucidate the functional significance of MMN and P3a responses to emotional cues in both auditory and visual modalities as neural indices of predictive coding that may contribute to individual behavioral differences in language comprehension and pragmatic skills.

## Figures and Tables

**Table 1:** The acoustic properties of each emotional prosody.

Emotions	Mean F0 (Hz)	Duration (ms)	Intensity Variation (dB)	HNR (dB)	Spectral Centroid (Hz)
Angry	216.71	646	11.15	9.22	1810.96
Happy	226.13	742	10.82	17.53	1052.92
Sad	180.42	822	10.18	19.31	408.79
Neutral	195.04	667	9.14	18.75	758.43

Note. The averaged values of all the words were used to report the mean fundamental frequency (F0), word duration, intensity variation, harmonics-to-noise ratio (HNR), and spectral centroid in each emotional prosody.

**Table 2:** Summary of the linear mixed-effect model using the amplitudes of MMN as the dependent variable. (** stands for *p* < 0.01; *** for *p* < 0.001).

Factor	Numerator df	Denominator df	*F*	*p*
Trial-level fixed factors				
Emotion (happy, sad, angry)	2	572	8.21	<0.001 ***
Region (anterior, central, parietal)	2	572	1.27	0.28
Laterality (left, mid, right)	2	572	0.59	0.55
Participant-level fixed factor				
Sex	1	22	0.001	0.97
Cross-level interaction				
Emotion * Sex	2	572	4.68	0.009 **

**Table 3:** Summary of the linear mixed-effect model using the amplitudes of P3a as the dependent variable. (*** stands for *p* < 0.001).

Factor	Numerator df	Denominator df	*F*	*p*
Trial-level fixed factors				
Emotion	2	572	25.67	<0.001 ***
Region	2	572	26.13	<0.001 ***
Laterality	2	572	1.54	0.21
Participant-level fixed factor				
Sex	1	22	0.49	0.49
Cross-level interaction				
Emotion * Sex	2	572	14.61	<0.001 ***

## Data Availability

Data will be made available upon reasonable request to the corresponding author. The data are not publicly available to comply with IRB regulation on data management.
